# Association between the NF-E2 Related Factor 2 Gene Polymorphism and Oxidative Stress, Anti-Oxidative Status, and Newly-Diagnosed Type 2 Diabetes Mellitus in a Chinese Population

**DOI:** 10.3390/ijms160716483

**Published:** 2015-07-20

**Authors:** Xia Wang, Hongxia Chen, Jun Liu, Yingying Ouyang, Di Wang, Wei Bao, Liegang Liu

**Affiliations:** 1 Department of Maternal and Child Health Care, School of Public Health, Shandong University, Jinan 250012, China; 2 Department of Nutrition and Food Hygiene and MOE Key Lab of Environment and Health, School of Public Health, Tongji Medical College, Huazhong University of Science and Technology, Wuhan 430030, China; E-Mails: liujunxju@sina.com (J.L.); xiongying0217@gmail.com (Y.O.); wad1983@126.com (D.W.); baowei0613@gmail.com (W.B.); 3 Institute of Biomedicine, Taihe Hospital, Hubei University of Medicine, Shiyan 442000, China; E-Mail: hustchenhx@163.com

**Keywords:** *NRF2*, oxidative stress, polymorphism, type 2 diabetes

## Abstract

Oxidative stress is a major risk factor in the onset and progression of type 2 diabetes mellitus (T2DM). NF-E2 related factor 2 (*NRF2*) is a pivotal transcription factor in oxidative stress related illnesses. This study included 2174 subjects with 879 cases of newly-diagnosed T2DM and 1295 healthy controls. Compared to individuals with the CC genotype, those with the AA genotype had lower total anti-oxidative capacity, superoxide dismutase, catalase, glutathione, glutathione peroxidase activity; and lower homeostasis model assessment of β-cell function index. Those with the AA genotype also had a higher malondialdehyde concentration and homeostasis model assessment of insulin resistance index values. The frequency of allele A was significantly higher in T2DM subjects (29.4%), compared to control subjects (26.1%; *p* = 0.019). Individuals with the AA genotype had a significantly higher risk of developing T2DM (OR 1.56; 95% CI 1.11, 2.20; *p* = 0.011), relative to those with the CC genotype, even after adjusting for known T2DM risk factors. Our results suggest that the *NRF2* rs6721961 polymorphism was significantly associated with oxidative stress, anti-oxidative status, and risk of newly-diagnosed T2DM. This polymorphism may also contribute to impaired insulin secretory capacity and increased insulin resistance in a Chinese population.

## 1. Introduction

Increases in the prevalence of diabetes have occurred internationally. It has been estimated that between 1980 and 2008, the number of adults with diabetes rose from 153 to 347 million [1]. Research suggests that oxidative stress is a major risk factor in the onset and progression of type 2 diabetes mellitus (T2DM) [2]. An oxidative environment may cause the development of impaired glucose tolerance, β-cell dysfunction, insulin resistance, and mitochondrial dysfunction, all of which can contribute ultimately to the diabetic disease state. Recent evidence also suggests that NF-E2 related factor 2 (*NRF2*) is a pivotal transcription factor of the antioxidant response in oxidative stress related illnesses [3,4,5].

*NRF2* is a member of the cap “n” collar (CNC) subfamily of basic leucine zipper transcription factors [6]. *NRF2* has highly conserved domains named Nrf2-erythroid-derived CNC homology protein homology (Neh) domains [7]. Among them, Neh1 domain is the CNC and basic leucine zipper domain, which interacts with partner proteins for heterodimerization [6]. The Neh3 domain, located at the extreme end of the carboxyl terminus of *NRF2*, is related to *NRF2* transactivation [8]. Neh4 and Neh5 cooperatively bind with the cyclic adenosine monophosphate response element binding protein-binding protein [9]. In the absence of *NRF2* structural data, it is not clear how Neh4 and Neh5, together with the Neh3 domains, exert trans-activation activity. The Neh2 domain, which is located in the N-terminus of *NRF2*, is a regulatory domain that responds to oxidative stress. Neh2 mediates binding with Kelch-like erythroid-derived protein with CNC homology-associated protein 1 (Keap1), and it negatively regulates *NRF2* function [10].

Keap1 was originally thought to be an actin-binding protein that represses the function of *NRF2* by sequestering *NRF2* in the cytoplasm [5,10]. Recently, Keap1 has also been identified as an adaptor protein between *NRF2* and Cullin3, a component of the E3 ligase complex. Under normal conditions, *NRF2* molecules may be subjected to continuous degradation by the proteasome [11,12]. When induced by oxidative stress derived from accumulation of reactive oxygen species (ROS) [13,14] or reactive nitrogen species [15,16,17], single or multiple reactive cysteine(s) in Keap1 can be modified. This conformation change causes *NRF2* to dissociate from Keap1. *NRF2* is quickly accumulated in the nucleus and elicits the antioxidant response by trans-activating the antioxidant response element (ARE) in the promoter region of many antioxidant genes [18,19]. The activation of *NRF2* is an important clue for the inducible expression of cytoprotective genes. The antioxidant enzymes encoded by these genes may play important roles in scavenging oxygen free radicals.

A few single nucleotide polymorphisms (SNPs) within the *NRF2* gene, such as rs7557529, rs6721961, rs35652124, rs2886161, rs1806649, have been reported [20,21,22]. Of these SNPs, SNP rs6721961, a variant of the *NRF2* gene in the upstream promoter region, has been showed to be associated with the risk of acute lung injury, an oxidative stress-mediated condition [21]. This human SNP, located in the middle of the ARE motif, can undermine the affinity of *NRF2* binding to the ARE. But the relationship between this polymorphism with β-cell function, insulin sensitivity, and the risk of T2DM is largely unknown.

Therefore, the objective of the present study was to evaluate the association between *NRF2* gene polymorphisms and susceptibility to newly-diagnosed T2DM in a relatively large Chinese population. We also evaluated the functional relevance of this polymorphism by measuring β-cell function, insulin sensitivity, oxidative stress, and anti-oxidative status among the study populations.

## 2. Results

### 2.1. Clinical and Biological Characteristics of Study Participants

Table 1 shows the general characteristics of the study participants. Both the patients and controls are in Hardy-Weinberg equilibrium (χ^2^ = 0.23, *p* = 0.63; χ^2^ = 3.23, *p* = 0.07, respectively). Participants with T2DM had a higher body mass index, a higher prevalence of family history of diabetes, more hypertension, more alcohol consumption, and higher levels of total cholesterol and triglyceride as compared to the control subjects. FPG and oral glucose tolerance test 2-h glucose levels, as well as hemoglobin A1c percentages, were significantly higher in patients with T2DM than in controls. It was worth noting that there was a significant difference for the age between T2DM and control groups (*p* < 0.001).

**Table 1:** Anthropometric and biochemical characteristics of the study populations.

Variable	NGT (*n* = 1295)	T2DM (*n* = 879)	*p* Value
Male (%)	59.00	57.23	0.116
Age (year)	45.54 ± 12.93	50.19 ± 11.29	<0.001
Body mass index (kg/m^2^)	22.77 ± 3.25	24.78 ± 3.34	<0.001
Hypertension (%)	19.41	45.30	<0.001
Drinker (%)	47.00	33.12	<0.001
Smoker (%)	46.53	37.44	<0.001
Regular exercise (%)	23.08	15.31	<0.001
Family history of diabetes (%)	12.81	18.82	<0.001
Fasting plasma glucose (mmol/L)	4.77 ± 0.64	9.81 ± 3.05	<0.001
OGTT2h (mmol/L)	6.44 ± 1.05	16.40 ± 3.81	<0.001
Fasting plasma insulin (μU/mL)	9.38 ± 6.10	10.41 ± 7.91	0.021
hemoglobin A1c (%)	5.68 ± 0.63	8.65 ± 2.38	<0.001
Triglycerides (mmol/L)	1.46 ± 1.12	2.09 ± 1.57	<0.001
Total cholesterol (mmol/L)	4.43 ± 0.90	4.73 ± 1.36	<0.001
HDL-C (mmol/L)	1.36 ± 0.60	1.48 ± 0.82	0.107
LDL-C (mmol/L)	2.59 ± 0.87	2.54 ± 1.21	0.662

Continuous variables are presented as means ± SD and categorical variables as numbers with percentage. Abbreviations: HDL-C, high density lipoprotein cholesterol; LDL-C, low density lipoprotein cholesterol; NGT, normal glucose tolerant; OGTT, oral glucose tolerance test; OGTT2h, 2-h post-glucose load; T2DM, type 2 diabetes mellitus.

### 2.2. Association of the NRF2 rs6721961 Polymorphism with Oxidative Stress and Anti-Oxidative Status

Table 2 presents β-cell function, insulin sensitivity, oxidative stress, and anti-oxidative status in relation to genotypes of rs6721961 polymorphism in the study populations. Compared to individuals with the CC genotype, those with the AA genotype had a significant decrease in plasma TAC levels (*p* = 0.025), CAT activity (*p* = 0.001), erythrocyte SOD activity (*p* = 0.042), GPx activity (*p* = 0.020), and GSH content (*p* = 0.042) and a significant increase in plasma MDA concentration (*p* = 0.005).

**Table 2:** Associations of β-cell function, insulin sensitivity, oxidative stress, and anti-oxidative status with genotypes of rs6721961 polymorphism in the study populations.

Variable	C/C	C/A	A/A	*p* (AA *vs*. CC)	*p* (AA *vs*.CA)
HOMA-IR	2.36 ± 0.06	2.53 ± 0.08	2.90 ± 0.19	0.003	0.048
HOMA-β	84.37 ± 1.59	86.98 ± 1.85	71.37 ± 4.05	0.005	0.001
TAC (U/mL)	9.23 ± 0.08	9.27 ± 0.09	8.73 ± 0.22	0.025	0.017
MDA (nmol/mL)	5.84 ± 0.11	6.02 ± 0.34	6.42± 0.26	0.005	0.019
SOD (U/gHb)	10,809.65 ± 64.64	10,602.73 ± 65.15	10,446.01 ± 118.61	0.042	0.049
CAT (KU/L)	38.14 ± 0.41	37.04 ± 0.44	34.41 ± 0.58	0.001	0.026
GPX (AU)	140.57 ± 1.06	139.63 ± 1.09	133.57 ± 3.61	0.020	0.047
GSH (mg/gHb)	20.10 ± 0.10	20.13 ± 0.12	19.49 ± 0.31	0.042	0.036

Abbreviations: HOMA-β, homeostasis model assessment of β-cell function index; HOMA-IR, homeostasis model assessment of insulin resistance index; CAT, catalase; GPX, glutathione peroxidase; GSH, glutathione; MDA, malondialdehyde; SOD, superoxide dismutase; TAC, total anti-oxidative capacity.

### 2.3. Association of NRF2 rs6721961 Polymorphism with β-Cell Function and Insulin Sensitivity

With regard to β-cell function and insulin sensitivity, as shown in Table 2, there were statistically significant differences in HOMA-β and HOMA-IR values. Individuals carrying the AA genotype had lower HOMA-β (AA 71.37 ± 4.05 *vs*. CC 84.37 ± 1.59, *p* = 0.005) and higher HOMA-IR values than did those with the CC genotype (AA 2.90 ± 0.19 *vs*. CC 2.36 ± 0.06, *p* = 0.003).

### 2.4. Association of NRF2 rs6721961 Polymorphism with Risk of Newly-Diagnosed Type 2 Diabetes Mellitus

Table 3 illustrates the genotype and allele frequencies of the rs6721961 polymorphism in the *NRF2* gene in the study populations. There were significant differences in the allelic frequency of the rs6721961 polymorphism between T2DM cases and controls. The frequency of allele A was significantly higher in T2DM subjects (29.4%), compared to NGT subjects (26.1%) (*p* = 0.019). The rs6721961 was associated with increased risk of diabetes. Individuals carrying the AA genotype had a significantly higher risk for developing T2DM (OR 1.77; 95% CI 1.26, 2.49; *p* = 0.011) relative to those with the CC genotype. This association remained statistically significant after adjusting for age, sex, body mass index, smoking, alcohol consumption, hypertension, family history of diabetes, and physical activity (OR 1.56; 95% CI 1.11, 2.20; *p* = 0.014).

**Table 3:** Genotype and allelic distributions of the rs6721961 polymorphism of the *NRF2* gene in participants.

Allele and Genotype	T2DM (%) (*n* = 879)	Controls (%) (*n* = 1295)	Crude OR (95% CI), *p*	Adjusted OR (95% CI), *p*
C/C	441 (50.2)	694 (53.6)	1	1
C/A	359 (40.8)	525 (40.5)	1.41 (1.05–1.89), 0.024	1.02 (0.85–1.23), 0.812
A/A	79 (9.0)	76 (5.9)	1.77 (1.26–2.49), 0.011	1.56 (1.11–2.20), 0.014
C	1241 (70.6)	1913 (73.9)	1	1
A	517 (29.4)	677 (26.1)	1.30 (1.13–1.51), 0.002	1.14 (0.99–1.31), 0.066

Adjusted for age, sex, body mass index, smoking, alcohol consumption, hypertension, family history of diabetes, and physical activity. Abbreviations: CI, confidence interval; OR, odds ratio; T2DM, type 2 diabetes mellitus.

### 2.5. Oxidative Stress and Antioxidant Status in the Study Population

Oxidative stress and anti-oxidative status in participants with and without diabetes is presented in Table 4. When compared with subjects with NGT, patients with T2DM had a significant increase in plasma MDA (*p* = 0.001) and a significant decrease in plasma TAC levels (*p* = 0.024) and CAT activity (*p* < 0.0001). Erythrocyte SOD activity (*p* = 0.002), GPx activity (*p* = 0.002), and GSH content (*p* = 0.040) were also significantly lower in patients with T2DM.

**Table 4:** Oxidative stress and anti-oxidative status in participants.

Variable	NGT (*n* = 1295)	T2DM (*n* = 879)	*p* Value
TAC (U/mL)	9.57 ± 0.17	8.86 ± 0.13	0.024
MDA (nmol/L)	5.79 ± 0.12	6.52 ± 0.18	0.001
SOD (U/gHb)	11,024.28 ± 299.13	9997.75 ± 164.01	0.002
GSH (mg/gHb)	20.87 ± 0.48	18.94 ± 0.29	0.040
CAT (KU/L)	41.71 ± 1.65	32.04 ± 1.04	<0.001
GPX (AU)	145.63 ± 4.32	129.97 ± 2.83	0.002

All abbreviations refer to Table 1 and Table 2.

## 3. Discussion

In this study, the rs6721961 polymorphism of the *NRF2* gene was found, which was consistent with previous reports by Yamamoto [23]. The frequency of allele A was significantly higher in newly-diagnosed T2DM subjects, compared to NGT subjects, indicating that there was an intrinsic linkage between *NRF2* genetic variants and the risk of T2DM. The present study investigated the effect of *NRF2* rs6721961 polymorphism on β-cell function and insulin sensitivity, oxidative stress and anti-oxidative status. The results also demonstrated that patients with newly-diagnosed T2DM had an increased free radical production and a reduced antioxidant capacity. Obviously, the age is a known validated risk factor of T2DM, since there was a significant difference for the age between T2DM and control groups in this study.

ROS include free radicals such as superoxide anion and hydroxyl radical, and non-radical hydrogen peroxide, which are constantly generated in aerobic organisms as byproducts of normal oxygen metabolism [24]. ROS serve as important physiological regulators in cell signaling at low concentrations. However, at higher concentrations, ROS can injure cellular macromolecules such as DNA, lipids, and proteins, which contributes to necrotic and apoptotic cell death [25]. To restrict the potential toxicity of ROS, cells have a well-developed antioxidant system. Low-molecular weight free radical scavengers included GSH, vitamin C, vitamin E, and complex enzymes contained CAT, GPx, SOD, *etc*.

The system can maintain redox balance or neutralize the toxic effects induced by ROS. However, when excessive ROS overwhelm the defense system or exceed its scavenging capability, oxidative stress may be ineluctable. Our study found that compared to individuals with the CC genotype, those with the AA genotype had a significant decrease in plasma CAT activity, erythrocyte SOD and GPx activity. Induction of these antioxidant enzymes is mediated largely by the transcription factor *NRF2* [26]. Decreased of antioxidants may cause an imbalance between prooxidants and antioxidants. This leads to cellular damage and ultimately T2DM [27]. Our data also supported the conclusion that decreased antioxidant activity could increase the risk of T2DM, as total TAC levels, CAT activity, erythrocyte SOD and GPx activity, and erythrocyte GSH content significantly decreased in T2DM patients.

Oxidative stress activates transcription of a variety of genes encoding anti-oxidative enzymes through a cis-acting sequence known as the ARE [28]. The ARE is initially found in promoter regions of genes encoding phase II detoxification enzymes and antioxidant proteins. Many studies show that *NRF2* is an essential element for regulation of the ARE [5]. *NRF2* rs6721961 is located in one of these ARE-like sites. One study by Marzec *et al*. examined the effects of this polymorphism on *NRF2*-DNA complex formation [21]. They found that *NRF2* binds less efficiently to ARE-like sequences that contain the −617 A allele, which decreases the boosting effect on its own transcriptional activity. By quantifying the *NRF2* mRNA in immortalized human lymphocytes, Suzuki *et al*. [29] found that the *NRF2* mRNA levels were significantly lower in A/A homozygotes than in C/A heterozygotes and C/C homozygotes by approximately 40%. The levels of expression of *tert*-butylhydroquinone-induced *NRF2* protein and *NQO1* mRNA were also lower in A/A homozygote than in C/C genotype lymphocytes [29]. These results indicated that the level of *NRF2* gene transcription is critical for the role of NRF2 in cytoprotection.

Moreover, hemin-inducible expression of heme oxygenase-1 was largely inhibited when the dominant mutant *NRF2* was over-expressed [30]. Up-regulation of heme oxygenase-1 may represent an attempt to minimize cellular injury [31]. Heme oxygenase-1 as an inducible stress protein can mitigate oxidative stress because of its potent anti-inflammatory, antioxidant, and anti-proliferative actions [32]. Our findings showed that polymorphisms in the *NRF2* genes were significantly associated with decreased antioxidant activity. It is possible that individuals with polymorphisms in *NRF2* were at increased risk of oxidative stress. Consistent with this hypothesis, targeted disruption of *NRF2* significantly reduced antioxidant capacity in mice and thus increased susceptibility to pro-oxidant and carcinogenic agents [33,34]. *NRF2* plays a key role in the protection of vertebrates against environmental stress by contributing to the inducible expression of detoxification and antioxidant enzymes [35].

There is sufficient evidence showing how *NRF2* affects β-cell function or insulin sensitivity. Recently, researchers highlighted the distinct roles that *NRF2* may play in pancreatic β-cell dysfunction that occurs in different stages of diabetes [36]. Pretreatment of MIN6 β cells with *NRF2* activators protects the cells from high levels of H_2_O_2_-induced cell damage [36]. Our results showed that individuals carrying the AA genotype had a lower HOMA-β and a higher HOMA-IR than did those with the CC genotype. This suggests that polymorphisms in the *NRF2* genes were associated with impaired β-cell function and increased insulin sensitivity. The polymorphisms in the *NRF2* genes might predispose individuals to impaired β-cell function, increased insulin sensitivity, and eventually T2DM. The pancreatic β cells express low levels of many antioxidant defense enzymes [37]. Oxidative stress is known to impair insulin secretion by pancreatic β cells. Insulin resistance is most often present in a large segment of the general population before the onset of diabetes [38]. Initially, β cells compensate for the prevailing insulin resistance to maintain plasma glucose concentration. When either the compensatory insulin secretory responses decrease or insulin resistance increases, or when both occur, it results in impaired glucose tolerance [39]. An increase in free fatty acid, insulin, and/or glucose levels can enhance ROS production and oxidative stress and activate stress-sensitive signal pathways. This, in turn, can deteriorate both insulin action and secretion and thereby accelerate the progression to overt T2DM [39].

Previous studies show a significant relationship between functional SNPs in *NRF2* and susceptibility to oxidative stress related illnesses such as acute lung injury [21]. It is likely related to the ability of transcription factor *NRF2* to modulate antioxidant and phase II enzyme genes that carry promoter AREs in their regulatory regions [40,41]. It may be postulated that individuals with polymorphisms in *NRF2* change basal expression of *NRF2* or the ability of *NRF2* to translocate from the cytoplasm to nuclear binding sites. Thus, individuals with polymorphisms in *NRF2* were at increased risk of oxidative stress and T2DM.

Accumulating lines of evidence have revealed that *NRF2* activation has also emerged as a hopeful target for the prevention of diabetic complications. In a mouse model of diabetes induced by methylglyoxal, treatment with resveratrol, which has been proposed as an effective treatment that helps lower the risk of developing complications of diabetes, markedly improved blood glucose level from the oral glucose tolerance test and promoted *NRF2* phosphorylation of the pancreas [42]. Also, short-term curcumin intervention has been shown to ablate diabetic kidney disease progress by activating *NRF2* anti-oxidative system and anti-inflammatory efficacies in patients with T2DM. Further, the natural antioxidants resveratrol may be useful in the treatment of type-2 diabetes by protecting against pancreatic cell dysfunction [43]. In the present study, the association between the rs6721961 polymorphism of the *NRF2* gene and an increased risk of T2DM was certainly well-founded. The results indicate an important role for *NRF2* in the development of T2DM. Because *NRF2* is critical to regulate antioxidant defense, polymorphisms that affect *NRF2* activity may have fundamental importance to T2DM. The present study provides direct support that *NRF2* has a central role in the oxidative stress response and T2DM. What we have found in the study has great significance in the treatment and management of type 2 diabetic patients. This risk allele may be useful as a clinical marker for identifying individuals who are susceptible to T2DM.

Oxidative stress may be increased in diabetes mellitus because of the increase in the production of oxygen free radicals and a deficiency in antioxidant defense mechanisms [4,5]. Many studies have examined oxidative stress markers and the status of antioxidants and antioxidant enzymes in diabetic patients [44,45,46]. However, the results were inconsistent. In the present study of a relatively large Chinese population, we found that patients with diabetes had a significant increase in plasma MDA and a significant decrease in plasma TAC and CAT activity, erythrocyte SOD and GPx activity, and erythrocyte GSH content. These findings demonstrated that T2DM had a strong association with oxidative stress, originating from decreased antioxidant potential and increased free radical production. Hyperglycemia can lead to an increase in oxidative stress markers such as membrane lipid peroxidation. The degree of lipid peroxidation in erythrocytes is directly proportional to glucose concentrations *in vitro* and blood glucose concentrations in diabetic patients [44]. Lipid peroxidation can generate large amounts of reactive products when free radicals attack of membrane lipids, which have been implicated in diabetes. MDA is a decomposition product of peroxidized polyunsaturated fatty acids and used widely as the marker of lipid peroxidation [45]. Glycation of anti-oxidative enzymes during hyperglycemia can damage cellular defense mechanisms, contributing to the development of oxidative stress and the progression of diabetes [46]. Thus, inhibition of enzymatic activity and a decrease in anti-oxidative enzymes caused by glycation are significantly contributors to the overall oxidative environment seen in diabetics.

There are some limitations in this study. First, an additional small-scale study is needed to determine the relationship between rs6721961 and the expression levels of *NRF2* and its representative target NQO1 mRNAs in lymphocytes of diabetic patients. These data will provide important information regarding the relationship between *NRF2* and onset of diabetes mellitus. In addition, we analyzed only one SNP rs6721961 and haplotypes of the promoter region only, which does not exclude any association of other regions around or within the gene. Further investigations, such as whole-gene sequencing, may be helpful for illuminating its role in T2DM. Furthermore, the role of potential confounders (such as resveratrol, which is abundant in red wine) were not evaluated in this study.

## 4. Experimental Section

### 4.1. Study Population

The study populations consisted of 879 cases with newly-diagnosed T2DM and 1295 normal glucose tolerant (NGT) controls. All cases were consecutively recruited from the outpatient clinics of the Department of Endocrinology at Tongji Medical College Hospital (Wuhan, China). Individuals were recruited from December 2004 to December 2007. Comparison controls were frequency-matched to patients by age and sex and were drawn from an unselected population that underwent routine health examinations in the same hospital. All participants met the respective diagnostic criteria recommended by the World Health Organization in 1999 [47]. For the study subjects, the inclusion criteria were as follows: no early history of diagnosed diabetes, no history of receiving pharmacologic treatment for diabetes, no clinically systemic diseases, and no other acute or chronic inflammatory diseases, cancer, and or acute respiratory infection. All subjects enrolled were of Chinese Han ethnicity. The study protocol was approved by the Ethics Committee of Tongji Medical College and written informed consent was obtained from all individuals.

### 4.2. Measurements of Biochemical Parameters, Lipid Peroxidation, and Antioxidant Status

Venous blood samples were obtained from all participants after an overnight fast. The samples were drawn from an antecubital vein into heparinized tubes. Plasma was used and retained for analysis of biochemical parameters, including fasting plasma glucose (FPG), fasting plasma insulin (FPI), hemoglobin A1c, and for estimation of total anti-oxidative capacity (TAC), catalase (CAT), and malondialdehyde (MDA). Subsequently, peripheral blood mononuclear cells were isolated by Ficoll-Hypaque density gradient centrifugation according to the manufacturer’s protocol. The erythrocyte pellets were collected and washed with 0.9% NaCl and then lysed in an appropriate volume of double-deionized water for the determination of hemoglobin and hemoglobin A1c, superoxide dismutase (SOD), and glutathione peroxidase (GPX) activity, as well as glutathione (GSH) concentration. Plasma and erythrocyte lysates were stored at −20 °C before analysis. 

Homeostasis model assessments of insulin resistance (HOMA-IR) and β-cell function (HOMA-β) were used to evaluate insulin sensitivity and insulin secretion, respectively. HOMA-IR = FPG (mmol/L) × FPI (µU/mL)/22.5, HOMA-β = 20 × FPI (µU/mL)/(FPG (mmol/L) − 3.5) [48]. The measure of plasma TAC was based on the ability of antioxidants in the samples to change Fe^3+^-tripyridyltriazine to Fe^2+^-tripyridyltriazine, a stable blue product proportional to the TAC, which was tested at 593 nm [49]. Plasma CAT activity was assayed by a method of Goth [50]. MDA as an index of lipid peroxidation was estimated by using the method described by Beuege and Aust [51]. The SOD activity in erythrocyte lysates was evaluated on the basis of its ability to inhibit the oxidation of hydroxylamine, as described previously [52]. Erythrocyte GPX activity was measured by the method described by Paglia and Valentine [53]. Erythrocyte GSH content was measured using the method described by Beutler *et al*. [54].

### 4.3. Genotyping

Genomic DNA was extracted from the leukocytes of fasting venous blood by using the phenol-chloroform method of DNA extraction [55]. The genotyping of SNP of the *NRF2* gene was done by using an allelic discrimination assay-by-design TaqMan method on ABI7900HT (Applied Biosystems, Foster City, CA, USA). Specifically, the primers of rs6721961 are as follows: Forward primer 1: CCCTGATTTGGAGGTGCAGAACC; Forward primer 2: GGGGAGATGTGGACAGCG; Reverse primer 1: GCGAACACGAGCTGCCGGA; Reverse primer 2: CTCCGTTTGCCTTTGACGAC. The primers and labelled oligonucleotide probes were designed and offered by Applied Biosystems. The TaqMan genotyping reaction was performed (50 °C for 2 min, 95 °C for 10 min, then followed by 40 cycles of 92 °C for 15 s and 60 °C for 1 min), and the endpoint fluorescent readings were done by ABI 7900HT data collection and analysis software version 2.2.1 (Applied Biosystems, Shanghai, China). Genotyping was performed as follows: 282, 113 bp for CC genotype; 282, 205, 113 bp for CA genotype; and 282, 205 bp for AA genotype. The genotyping error rate was examined by randomly re-genotyping 10% of the samples as blind duplicates and the concordance rate was 100%.

### 4.4. Statistical Analyses

Descriptive statistics in the clinical and laboratory characteristics of healthy controls and patients with T2DM were calculated for the study subjects. Differences between diabetes cases and controls were tested by one-way analysis of variance, followed by Chi-square (categorical variables) or Student’s *t* test (continuous variables). For analysis of the gene polymorphism, allelic repeats were divided into subgroups by their distributions. Differences in allelic and genotypic frequencies of the gene polymorphisms in healthy controls and patients with T2DM were compared by Chi-square test, which was also used to evaluate Hardy-Weinberg equilibrium for each individual locus. For analysis of linkage disequilibrium, the linkage disequilibrium coefficient and the correlation coefficient were estimated by using the LDA software program [56]. A *p* value < 0.05 was considered statistically significant.

We used logistic regression analysis to assess the association of diabetes events with the specific polymorphism. Odds ratios and 95% confidence intervals were adjusted for known risk factors for T2DM, including age, body mass index, sex, smoking, alcohol consumption, hypertension, family history of diabetes, and physical activity. Statistical analyses were performed using SPSS for windows software version 20.0 (SPSS Inc., Chicago, IL, USA).

## 5. Conclusions

The risk allele of rs6721961 in the *NRF2* gene is associated with oxidative stress, reduced anti-oxidative status, and increased risk of newly-diagnosed T2DM. This polymorphism may also contribute to impaired insulin secretory capacity and increased insulin resistance in a Chinese population. Understanding how the *NRF2* genotype modulates oxidative stress and the status of insulin action and insulin secretion, as well as how *NRF2* affects the risk of T2DM, may aid the design of new therapeutic approaches for the prevention and treatment of T2DM.
